# Identification of Chrysanthemum (*Chrysanthemum morifolium*) Self-Incompatibility

**DOI:** 10.1155/2014/625658

**Published:** 2014-01-27

**Authors:** Fan Wang, Feng-Jiao Zhang, Fa-Di Chen, Wei-Min Fang, Nian-Jun Teng

**Affiliations:** ^1^College of Horticulture, Nanjing Agricultural University, Nanjing 210095, China; ^2^Jiangsu Province Engineering Lab for Modern Facility Agriculture Technology & Equipment, Nanjing 210095, China

## Abstract

There has been a heated argument over self-incompatibilityof chrysanthemum (*Chrysanthemum morifolium*) among chrysanthemum breeders. In order to solve the argument, we investigated pistil receptivity, seed set, and compatible index of 24 chrysanthemum cultivars. It was found that the 24 cultivars averagely had 3.7–36.3 pollen grains germinating on stigmas at 24 hours after self-pollination through the fluorescence microscope using aniline blue staining method. However, only 10 of them produced self-pollinated seeds, and their seed sets and compatible indexes were 0.03–56.50% and 0.04–87.50, respectively. The cultivar “Q10-33-1” had the highest seed set (56.50%) and compatible index (87.50), but ten of its progeny had a wide range of separation in seed set (0–37.23%) and compatible index (0–68.65). The results indicated that most of chrysanthemum cultivars were self-incompatible, while a small proportion of cultivars were self-compatible. In addition, there is a comprehensive separation of self-incompatibility among progeny from the same self-pollinated self-compatible chrysanthemum cultivar. Therefore, it is better to emasculate inflorescences during chrysanthemum hybridization breeding when no information concerning its self-incompatibility characteristics is available. However, if it is self-incompatible and propagated by vegetative methods, it is unnecessary to carry out emasculation when it is used as a female plant during hybridization breeding.

## 1. Introduction

Chrysanthemum (*Chrysanthemum morifolium*) with a high ornamental value is one of the ten most popular traditional flowers in China and one of the four most popular cut flowers in the world; therefore this flower occupies a very important position in the world flower industry [1–4]. It is estimated that there are more than 20,000 chrysanthemum cultivars in the world and about 7,000 cultivars in China. Over 90% of them were produced through traditional hybridization breeding technique, and many new chrysanthemum cultivars are now being developed by this technique each year [4–6]. Although the traditional hybridization breeding method has played a key role in developing chrysanthemum cultivars by now, there has been a heated argument over necessity of inflorescence emasculation, a very complex and laborious process, during chrysanthemum hybridization breeding among chrysanthemum breeders [5–8]. Some breeders think that it is necessary to emasculate chrysanthemum inflorescences before pollination during chrysanthemum hybridization breeding, because they believe that chrysanthemum is self-compatible. In contrast, some other chrysanthemum breeders feel that it is unnecessary to make inflorescences emasculated during chrysanthemum hybridization breeding, as they think that chrysanthemum is a self-incompatible flower.

Emasculation of chrysanthemum inflorescences is a very complex and time-consuming process, as chrysanthemum inflorescence structure is not suitable for emasculation [3–5, 9]. Each chrysanthemum inflorescence consists of 20–30 peripheral ray florets with only pistil and 100–200 small central disk florets with both pistil and stamen [3, 4, 9]. If inflorescences need to be emasculated during chrysanthemum hybridization, all the small central disk florets should be completely removed by hands with tweezers at around 3 days before anthesis. Meanwhile, the upper parts (tubiform petals) of all peripheral ray florets should be entirely cut off by scissors until the stigmas are visible. Then, the emasculated inflorescences are bagged [3]. Thus, the emasculation of chrysanthemum inflorescences not only is very laborious but also sometimes causes injuries to the inflorescences, as a consequence resulting in low breeding efficiency. In addition, the emasculation process significantly reduces the number of florets that can be used for production of hybrid seeds [9, 10]. For example, if chrysanthemum is self-incompatible, the small central disk florets can be also used for production of hybrid seeds, because it is unnecessary to remove them. Therefore, it is very urgent and necessary to reveal if chrysanthemum is self-incompatibility or not.

According to our knowledge, there have been no studies systematically investigating self-incompatibility of chrysanthemum so far. We therefore carried out a systematical investigation on pistil receptivity, seed set, and compatible index of 24 chrysanthemum cultivars with high ornamental values in this study. In addition, we also examined pistil receptivity, seed set, and compatible index of some progeny of one chrysanthemum cultivar. The purpose of this study is to solve the above-mentioned argument among chrysanthemum breeders through identifying chrysanthemum self-incompatibility and related cellular mechanisms. In addition, we also hope that the expected results will provide valuable information for increasing breeding efficiency during chrysanthemum hybridization breeding in the future.

## 2. Materials and Methods

### 2.1. Experimental Materials

Twenty-four chrysanthemum cultivars with high ornamental values were used as materials in this study. They were grown in the Chrysanthemum Germplasm Resource Preserving Center, Nanjing Agricultural University, China (32°05′N, 118°90′E).

### 2.2. Determination of Selfing Seed Set and Compatible Index through Selfing Experiment

In order to get some information on seed set and compatible index of the 24 chrysanthemum cultivars, we first carried out selfing experiment. The selfing experiment was performed in early November 2011. Twenty to thirty inflorescences were randomly selected from each cultivar and then bagged with waterproof and breathable paper bags at around 3 days before anthesis. Cultivars' names, date, and the names of researchers were marked on the vegetable parchment tied with each inflorescence. Although the paper bags were waterproof and breathable, relative humidity sometimes was much higher inside than outside bags. Therefore, a small hole was made from each bag to prevent mildew. After two weeks, all the bags were removed from the inflorescences, because they had lost pistil receptivity. Around two months later (in the middle of January 2012), the inflorescences were cut off from plants for seed collection. After that, seed set (the average seed number per inflorescence/the average floret number per inflorescence) and compatible index (the seed number of all inflorescences/the number of all inflorescence) were then counted.

### 2.3. Artificial Self-Pollinated Experiment

Because it was difficult to know the exact dates of anther dehiscence and pollen dispersal in the selfing experiment, it was very difficult to determine pollen-stigma interaction or pistil receptivity. We therefore did artificial self-pollinated experiment, as the pollination time can be controlled. In the artificial self-pollinated experiment, five inflorescences were randomly selected from each cultivar at around 3 days before anthesis, then emasculated, and bagged. Around three days later when the shape of pistils became Y, it meant that the pistils had strong receptivity [3, 5]. At this time, artificial self-pollinated experiment was performed with fresh mature pollen grains that were collected from mature inflorescences on the same plant or other plants of the same cultivar. After artificial pollination, the inflorescences were bagged again.

### 2.4. Determination of Pistil Receptivity

Because our previous study indicated that the number of germinated pollen grains on chrysanthemum stigmas reached the peak at 24 h after artificial pollination, we thus here collected the self-pollinated inflorescences at 24 h after pollination in the artificial self-pollinated experiment. The self-pollinated inflorescences were cut off from each cultivar and immediately fixed and stored in FAA solution. The pistils were stripped out, softened in 1 mol/L NaOH solution for 8 h at room temperature, then washed with distilled water one time, and immersed in aniline blue staining fluid (0.1 mol/L K_3_PO_4_ + 18% glycerol) for one to two hours in the dark [1, 11]. After that, the pistils were placed on the glass slides and gently pressed with coverslips. Then, the germination behaviors of pollen grains on stigmas and the growth of pollen tubes were observed under a fluorescence microscope (Zeiss Axioskop 40). Good representative images were captured with an Axiocam MRC camera. For each cultivar, 30 pistils were observed and the numbers of germinated pollen grains on each stigma were recorded.

### 2.5. Examination of Embryo Development after Chrysanthemum Selfing

Because low seed set during selfing experiments may be caused by two factors, that is, self-incompatibility (pistil receptivity or pollen-stigma interaction) and embryo abortion, we examined embryo development after chrysanthemum selfing for the purpose of ruling out the possibilities that low seed set was caused by embryo abortion. At two weeks after the inflorescences were bagged in the selfing experiment, five inflorescences were sampled from each of 24 cultivars and immediately fixed in FAA solution (95% ethanol : distilled water : formalin : glacial acetic acid = 63 : 27 : 5 : 5) until use. Ovaries were collected form the florets of each inflorescence and subjected to dehydration through a graded series of ethanol solutions and then immersed in paraffin wax. After that, sections were cut to a thickness of 8–10 *μ*m and stained with Heidenhain's hematoxylin and then observed and photographed under an Olympus BX41 microscope [1, 3]. Because the cultivar “QX-118” did not produce any selfing seeds and there were a very high number of pollen grains germinated on its stigmas at 24 h after artificial pollination, embryo development of this cultivar was in particular investigated.

### 2.6. Investigation on Self-Incompatibility of “Q10-33-1”s Progeny

The cultivar, “Q10-33-1,” produced a lot of seeds after selfing in 2011, indicating that this cultivar is self-compatible. However, we did not know if its progeny inherited its characteristics of self-compatibility. We therefore also investigated seed set, compatible index, and pistil receptivity of 10 “Q10-33-1”s progeny in 2012. In the middle of March 2012, the selfing seeds of 9 cultivars were sown in a 1 : 2 (v/v) mixture of vermiculite and garden soil in the greenhouse. The day/night temperature regime and the relative humidity were around 25/18°C and 60–80%, respectively [2]. Two weeks later, seed germination rate was calculated. At 5 weeks after sowing, the seedlings at 6-leaf stage were transplanted to the Chrysanthemum Germplasm Resource Preserving Center, Nanjing Agricultural University, China. From early November 2012, seed set, compatible index, and pistil receptivity of 10 “Q10-33-1”s progeny were investigated according to the above-mentioned methods.

### 2.7. Statistical Analysis

The data were analyzed using the SPSS software 16.0 (SPSS Inc, Chicago, Ill, USA), and were shown as mean ± standard deviation.

## 3. Results

### 3.1. Selfing Seed Set and Compatible Index of 24 Chrysanthemum Cultivars

There are large differences in selfing seed set and compatible index among the 24 chrysanthemum cultivars (Table 1). Among them, 15 cultivars did not produce any seeds after selfing. As a consequence, their seed set and compatible index were 0, indicating that the 15 cultivars were completely self-incompatible. Although the other 9 cultivars produced selfing seeds, seed sets and compatible indexes of five cultivars (“QX-081,” “QX-006,” “QX-003,” “Q10-33-2,” and “Nannongxiangbin”) were very low, much less than 0.5%, showing that the five cultivars were nearly self-incompatible. For “Nannonghongcheng,” “QX-001,” and “Nannongjinhe,” their seed sets and compatible indexes ranged from 0.50% to 1.73%, demonstrating that the three cultivars were partly self-compatible. For “Q10-33-1,” it was a highly self-compatible cultivar, as this cultivar had a very high seed set (56.50%) and compatible index (87.50%). The results suggested that most of chrysanthemum cultivars are self-incompatible or nearly self-incompatible, and only a small proportion of cultivars are self-compatible.

### 3.2. The Germination Behavior of Pollen Grains on Stigmas of 24 Chrysanthemum Cultivars

**Figure 1:**
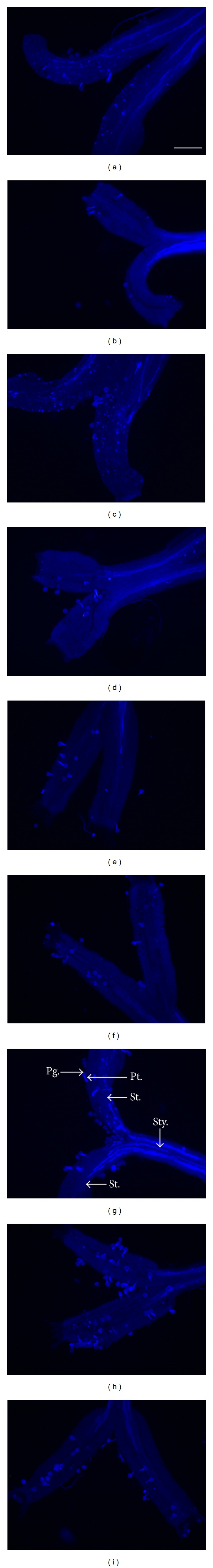
Germination behaviour of pollen grains on chrysanthemum stigmas at 24 hours after self-pollination. ((a), (b), and (c)) Three representative cultivars (“QX-081,” “Nannonghonghe,” “QX-006 2f-3,” and “Nannongxiangbin”) with low number of pollen grains germinating on per stigma. ((d), (e), and (f)) Three representative cultivars (“Nannongxiangbin”, “Q10-6-9,” and “Q10-17-3”) with medium number of pollen grains germinating on per stigma. ((g), (h), and (i)). Three representative cultivars (“QX-097,” “Q10-33-1,” and “QX-149”) with high number of pollen grains germinating on per stigma. Abbreviations: Pg.: pollen grain, Pt.: pollen tube, St.: stigma, and Sty.: style. All the figures have the same bar (100 *μ*m).

The average number of pollen grains germinating on each stigma greatly varied among the 24 chrysanthemum cultivars (Table 1; Figures 1(a)–1(i)). The lowest number was 3.7 in “Q10-22-2” and the highest was 36.3 in “Nannongjinhe.” In addition, the number of pollen grains germinating on each stigma was not positively proportional to selfing seed set and compatible index. For example, “Q10-33-1” had the highest seed set and compatible index among these cultivars, but the average number of pollen grains germinating on each stigma was 21.9 that was lower than those of “QX-149,” “QX-002,” “QX-118,” and “Nannongjinhe”. Many pollen grains germinated on stigmas of “QX-149,” “QX-002,” “QX-118,” but their seed sets and compatible indexes were 0. One of the possible reasons is that the pollen tubes of the three cultivars germinated abnormally and failed to transfer sperms into embryo sacs for double fertilization. In contrast, although “QX-001” had only 8.6 pollen grains that germinated on each stigma at 24 h after artificial pollination, it still produced some selfing seeds. Therefore, self-compatible cultivars usually have high pistil receptivity or the average number of pollen grains germinating on each stigma. However, if chrysanthemum cultivars have high pistil receptivity, it does not mean that the cultivars are self-compatible.

### 3.3. Embryogenesis after Selfing

**Figure 2:**
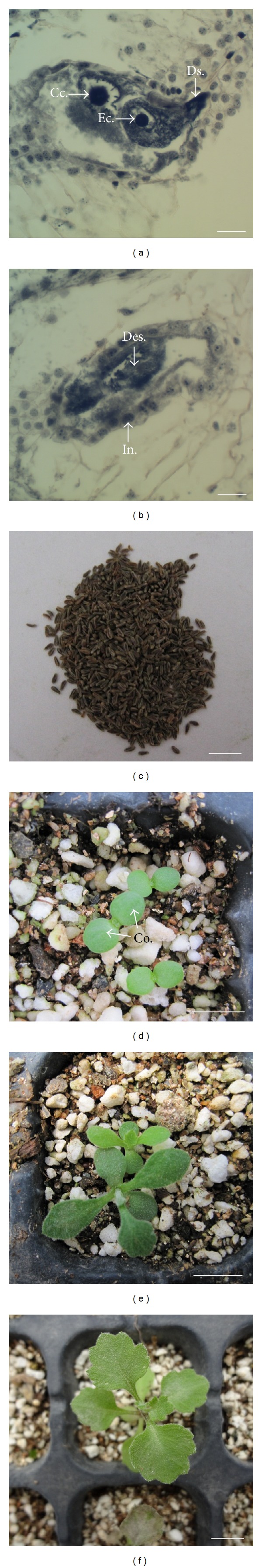
Ovary anatomy, seeds, and seedlings of chrysanthemum. (a) An unfertilized embryo sac at two weeks after self-pollination. (b) A degenerative embryo sac at two weeks after self-pollination. (c) Self-pollinated seeds of “Q10-33-1.” ((d), (e), and (f)) Progeny of self-pollinated “Q10-33-1” at different growth stages. Abbreviations: Cc.: central cell, Co.: cotyledon, Des.: degenerative embryo sac, Ds.: degenerative synergid, Ec.: egg cell, and In.: integument. Bars: 100 *μ*m ((a) and (b)) and 1 cm ((c)–(f)).

Our previous studies indicated that chrysanthemum embryo development usually reaches the stage of globular embryo at around 10–15 d after pollination if double fertilization occurs normally [1, 3]. However, we did not observe any embryos from the inflorescences that were collected at two weeks after they were bagged in the selfing experiment. Instead, we found that double fertilization did not happen in chrysanthemum embryo sacs, as the embryo sacs were degenerating and the degradation residues of synergids, egg cells, or central nuclei could be easily observed in the embryo sacs (Figures 2(a) and 2(b)). Such results ruled out the possibilities that low seed set resulted from embryo abortion and confirmed self-incompatibility was the reason that chrysanthemum cultivars did not produce any selfing seeds.

### 3.4. Germination of Selfing Seeds and Their Growth

Selfing seeds began to germinate at around 5 days after sowing, and most of seeds germinated within two weeks after sowing (Figures 2(c) and 2(d)). Thereafter, no seeds germinated and germination rate of selfing seeds for each cultivar was calculated. The results indicated that selfing seeds from “QX-081,” “QX-006,” “QX-003,” and “Nannongxiangbin” did not germinate (Table 2). The results combined with their seed sets and compatible indexes firmly confirmed that the four cultivars were also self-incompatible. Seed germination rates for other five cultivars (“Q10-33-2,” “Nannonghongcheng,” “QX-001,” “Nannongjinhe,” and “Q10-33-1”) ranged from 23.9% to 66.7%, further confirming they were self-compatible. Most of seedlings reached 4-leaf stage and 6-leaf stage at around 3 and 5 weeks after sowing, respectively (Figures 2(e) and 2(f)). Then progeny of “Q10-33-1” at 6-leaf stage were transplanted to the field for investigation on their self-incompatibility.

### 3.5. Self-Incompatibility of “Q10-33-1”s Progeny

**Figure 3:**
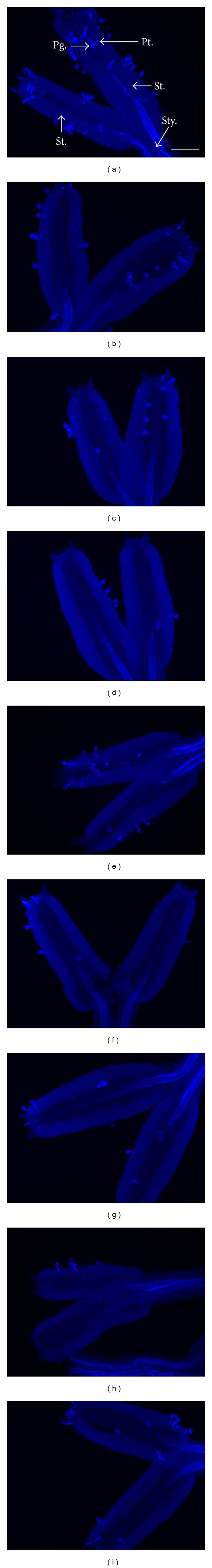
Germination behaviour of pollen grains on stigmas of nine “Q10-33-1”s progeny at 24 hours after self-pollination. ((a)–(i)) Q10-33-1*①*–*⑨*. Abbreviations: Pg.: pollen grain, Pt.: pollen tube, St.: stigma, and Sty.: style. All the figures have the same bar (100 *μ*m).

“Q10-33-1” was high self-compatible, while there was comprehensive separation of self-incompatibility characteristics among its progeny. For example, seed set and compatible index of “Q10-33-1” were 56.50% and 87.50%, respectively (Table 1). However, seed set and compatible index of ten “Q10-33-1”s progeny were 0–37.23% and 0–68.65%, respectively (Table 3). The average number of pollen grains germinating on each stigma among the ten progeny ranged from 1.40 to 30.43, which was nearly positively proportional to seed set and compatible index (Table 3; Figures 3(a)–3(i)). The results suggested that self-compatible chrysanthemum cultivars can produce both self-compatible and self-incompatible progeny, which is the reason why there is wide separation of self-incompatibility among chrysanthemum. The comprehensive separation of self-incompatibility characteristics among selfing progeny may be mainly attributed to complex genetic background of chrysanthemum cultivars that are allohexaploid species with an average chromosome number of 54.

## 4. Discussion

Self-incompatibility is an important genetic mechanism to prevent plant inbreeding through preventing self-fertilization [12–15]. When a viable pollen grain of a plant with self-incompatibility lands on a stigma of the same plant or another plant with a similar genotype, it fails to germinate or pollen tube growth is inhibited, and consequently fertilization does not germinate and no seeds are produced [12]. Self-incompatibility has been found in many families, such as Solanaceae, Papaveraceae, Rosaceae, Scrophulariaceae, Brassicaceae, Asteraceae, and Convolvulaceae [12, 16–21]. In the present study, although 15 chrysanthemum cultivars did not produce selfing seeds, it did not mean that they were self-incompatible. Because seed production is closely related to the events including pollen germination, pollen tube growth, ovule fertilization, and embryo development, any abnormal events can result in a total failure in seed production [22–25]. Therefore, in order to rule out the possibilities that embryo abortion was not a factor leading to the failure in seed production of these chrysanthemum cultivars, we investigated their embryo development at two weeks after the inflorescences were bagged. The results indicated that the ovules were not fertilized at all and the embryo sacs underwent degradation, and thus embryo abortion was not the reason for the failure in production of selfing seeds. In other words, self-incompatibility was the reason of failure in production of selfing seeds, and these cultivars were confirmed to be self-incompatible.

Pollen germination and pollen tube growth on stigmas are closely related to self-incompatibility characteristics [12, 22, 26]. When a viable pollen grain of a plant reaches a stigma of the plant and does not germinate, the plant can be easily regarded to be self-incompatible. For example, no viable pollen grains germinated on stigmas of *Viburnum macrocephalum* f. *keteleeri* after self-pollination, but the plant produced many seeds after pollination with fresh viable pollen grains from another plant; thus this species was considered to be self-incompatible [27, 28]. However, it is difficult to identify whether a plant is self-incompatible or not immediately if a pollen grain germinates on its stigma, as sometimes abnormal pollen tube growth is an indicator of self-incompatibility and it is difficult to examine behaviors of pollen tube growth in some species [22, 29]. Such a case happened in chrysanthemum. In this study, it was found there was no close relationship between self-incompatibility and the number of pollen grains germinating on chrysanthemum stigma, as selfing seed set of the 24 chrysanthemum cultivars was not positively proportional to the number of pollen grains that germinated on stigmas. For instance, “QX-118” had a higher number of pollen grains that germinated on stigmas compared with “Q10-33-1,” while “QX-118” did not produce selfing seeds and selfing seed set of “Q10-33-1” was as high as 56.5% (Table 1). Because it was difficult to discriminate morphological features of pollen tubes on stigmas between “Q10-33-1” and “QX-118,” a possible reason for this discrepancy is that the growth of “QX-118” pollen tubes was abnormal or inhibited, and few pollen tubes could grow toward the embryo sac along the transmitting tissues of style, and as a consequence the ovules were not fertilized. In contrast, “Q10-33-1” pollen tubes grew normally and transferred the sperms into embryo sacs for fertilization.

There has been a heated argument over self-incompatibility of chrysanthemum among chrysanthemum breeders. Some of them think that chrysanthemum is completely self-incompatible, while the others believe that chrysanthemum is self-compatible [5–7]. The results of the current study indicated there was a wide separation of self-incompatibility in chrysanthemum. Most of chrysanthemum cultivars were self-incompatible, but a small portion of cultivars were self-compatible, demonstrating that chrysanthemum self-incompatibility is cultivar dependent. Similar phenomena were also found in some other species including Japanese apricot, peach, and cabbage [30–32]. Taken Japanese apricot as an example, most of cultivars are self-incompatible, and some cultivars are self-compatible [30]. The results presented here provide evidence that chrysanthemum self-incompatibility is cultivar dependent. Therefore, chrysanthemum breeders are suggested to emasculate inflorescences during chrysanthemum hybridization breeding in the future, though emasculation is very laborious and sometime causes injuries to inflorescences. However, if information shows that chrysanthemum cultivars are self-incompatible, then it is unnecessary to perform emasculation when these cultivars are used as female plants. The results in this study show that fifteen cultivars without self-pollinated seeds and four cultivars without germination of their self-pollinated seeds are self-incompatible (Tables 1 and 3). When the nineteen cultivars are used as maternal plants in hybridization breeding, they need not be emasculated.

Self-pollinated seeds of four chrysanthemum cultivars failed to germinate, many self-pollinated seeds of five cultivars did not germinated, and germination rate of “Q10-33-1” seeds was only 23.9%. Tang once considered that some chrysanthemum “seeds” were not the real seeds, because they did not contain embryos. Such “seeds” were possibly produced from ovary tissues after they were stimulated by pollen-pistil interaction [33]. Another possible reason is that some self-pollinated seeds did not contain normal embryos, for some previous studies showed that embryos frequently degenerated at different development stages in chrysanthemum hybridization breeding [1, 3, 34]. We recently examined anatomical features of some chrysanthemum self-pollinated seeds and found that some of them really did not contain any embryos, and some others had abnormal embryos in morphology (data no shown). Such results support the two explanations mentioned above. In addition, it was found that self-incompatibility separated widely among the selfing progeny; some progeny were self-incompatible while some were self-compatible. A possible explanation for this phenomenon is that chrysanthemum is an allohexaploid species with an average chromosome number of 54, and its genetic background is very complex [4, 5]. Because self-incompatibility of plants is genetically controlled by a multiallelic S locus [12], it is more possible that there is a wide separation in self-incompatibility characteristics among chrysanthemum selfing progeny. There have been no studies reporting molecular mechanism of chrysanthemum by now, and no information is available on chrysanthemum pollen self-incompatibility determinant and pistil S locus component; therefore it is impossible now to explain the underlying reason of the separation in self-incompatibility among chrysanthemum selfing progeny.

In summary, we carried out a systematical investigation on pistil receptivity, seed set, and compatible index of 24 chrysanthemum cultivars and ten selfing progeny of one cultivar in the current study. Two finding are worth noting. The first one is that most of chrysanthemum cultivars were self-incompatible, and a small proportion of chrysanthemum cultivars were self-compatible. The second one is that there is a comprehensive separation of self-incompatibility among progeny from the same self-pollinated self-compatible chrysanthemum cultivar. The findings suggest that emasculation should be done during chrysanthemum hybridization breeding when information on its self-incompatibility characteristics is unavailable. However, if a chrysanthemum cultivar is self-incompatible, and is reproduced by vegetative propagation, it is unnecessary to do emasculation when used as a female plant during hybridization breeding.

## Figures and Tables

**Table 1:** Pistil receptivity, seed set, and compatible index of 24 cultivars.

Cultivar	Number of pollen grains germinating per stigma	Seed set (%)	Compatible index
“Q10-22-2”	3.7 ± 1.1	0	0
“Nannonghonghe”	6.0 ± 1.2	0	0
“Q07-26-3”	6.7 ± 1.6	0	0
“QX-113”	9.0 ± 1.3	0	0
“Q08-4-2”	9.1 ± 1.3	0	0
“QX-004”	9.8 ± 1.4	0	0
“260Shanghai”	13.2 ± 2.4	0	0
“Q10-6-9”	15.0 ± 1.3	0	0
“Q10-17-3”	17.0 ± 1.7	0	0
“QX-148”	17.9 ± 1.5	0	0
“QX-145”	19.8 ± 1.7	0	0
“QX-097”	20.6 ± 2.3	0	0
“QX-149”	22.8 ± 2.6	0	0
“QX-002”	24.0 ± 2.9	0	0
“QX-118”	29.6 ± 2.6	0	0
“QX-081”	5.7 ± 1.3	0.03 ± 0.00	0.04 ± 0.00
“QX-006”	7.8 ± 1.3	0.03 ± 0.00	0.04 ± 0.00
“QX-003”	10.5 ± 1.6	0.07 ± 0.03	0.12 ± 0.03
“Q10-33-2”	10.2 ± 1.5	0.08 ± 0.02	0.13 ± 0.02
“Nannongxiangbin”	12.3 ± 2.3	0.18 ± 0.05	0.22 ± 0.06
“Nannonghongcheng”	9.1 ± 1.5	0.50 ± 0.08	0.97 ± 0.12
“QX-001”	8.6 ± 1.4	0.71 ± 0.11	1.43 ± 0.09
“Nannongjinhe”	36.3 ± 3.3	1.24 ± 0.32	1.73 ± 0.24
“Q10-33-1”	21.9 ± 2.0	56.50 ± 4.73	87.50 ± 6.92

Values given are mean ± standard deviation.

**Table 2:** Germination rate of selfing seeds.

Cultivars	Seed germination rate (%)
“QX-081”	0
“QX-006”	0
“QX-003”	0
“Q10-33-2”	66.7 ± 8.3
“Nannongxiangbin”	0
“Nannonghongcheng”	57.1 ± 6.5
“QX-001”	66.7 ± 7.2
“Nannongjinhe”	27.5 ± 4.7
“Q10-33-1”	23.9 ± 4.3

Values given are mean ± standard deviation.

**Table 3:** Pistil receptivity, seed set, and compatible index of ten “Q10-33-1”s progeny.

Progeny	Number of pollen grains germinating per stigma	Seed set (%)	Compatible index
“Q10-33-1*①*”	30.43 ± 2.21	37.23 ± 3.17	68.65 ± 5.42
“Q10-33-1*②*”	11.73 ± 1.60	36.32 ± 2.65	55.35 ± 3.86
“Q10-33-1*③*”	8.57 ± 1.04	26.77 ± 2.24	40.48 ± 3.51
“Q10-33-1*④*”	5.33 ± 1.24	7.97 ± 0.93	14.64 ± 1.12
“Q10-33-1*⑤*”	5.47 ± 1.48	5.35 ± 0.71	4.25 ± 0.54
“Q10-33-1*⑥*”	1.40 ± 0.62	0.26 ± 0.04	0.41 ± 0.06
“Q10-33-1*⑦*”	2.73 ± 0.78	0.06 ± 0.02	0.08 ± 0.03
“Q10-33-1*⑧*”	1.47 ± 0.68	0.03 ± 0.00	0.06 ± 0.00
“Q10-33-1*⑨*”	4.73 ± 1.46	0.02 ± 0.00	0.03 ± 0.00
“Q10-33-1*⑩*”	7.20 ± 1.42	0.00	0.00

Values given are mean ± standard deviation.
